# Mosaic complete tetrasomy 21 in a fetus with complete atrioventricular septal defect and minor morphological variations

**DOI:** 10.1002/mgg3.895

**Published:** 2019-09-07

**Authors:** Vincent Gatinois, Nicole Bigi, Eve Mousty, Jean Chiesa, Yuri Musizzano, Anouck Schneider, Geneviève Lefort, Lucile Pinson, Jean‐Baptiste Gaillard, Clémence Ragon, Marie‐Josée Perez, Magali Tournaire, Patricia Blanchet, Carole Corsini, Emmanuelle Haquet, Patrick Callier, David Geneviève, Franck Pellestor, Jacques Puechberty

**Affiliations:** ^1^ Laboratoire de Génétique Chromosomique Hôpital Arnaud de Villeneuve CHU de Montpellier Montpellier France; ^2^ Service de Génétique Clinique Département de Génétique Médicale Maladies Rares et Médecine Personnalisée Hôpital Arnaud de Villeneuve CHU de Montpellier Montpellier France; ^3^ Département de Gynécologie‐Obstétrique Hôpital Carémeau CHU de Nîmes Nîmes France; ^4^ Laboratoire de Cytologie Clinique et Cytogénétique Hôpital Carémeau CHU de Nîmes Nîmes France; ^5^ Laboratoire d'Anatomie et Cytologie Pathologique Hôpital Gui‐de‐Chauliac CHU de Montpellier Montpellier France; ^6^ Laboratoire de Génétique Moléculaire et Cytogénétique Hôpital du Bocage CHU de Dijon Dijon France

**Keywords:** atrioventricular septal defect, Down syndrome, Mosaicism, prenatal diagnosis, tetrasomy 21, trisomy 21

## Abstract

**Background:**

Tetrasomy 21 is a very rare aneuploidy which could clinically resemble a Down syndrome. It was most often described in its partial form than complete. We report the prenatal, pathological and genetic characteristics of a fetus with mosaic complete tetrasomy 21. This is the second well‐documented description of a complete tetrasomy 21 in the literature.

**Methods:**

Prenatal and fetal pathological examinations, cytogenetic and molecular analyses were performed to characterize fetal features with tetrasomy 21.

**Results:**

Prenatal ultrasound examination revealed an isolated complete atrioventricular septal defect with normal karyotype on amniotic fluid. After termination of pregnancy, clinical examination of the fetus evoked trisomy 21 or Down syndrome. Chromosomal microarray analysis and FISH on lung tissue showed a mosaicism with four copies of chromosome 21 (tetrasomy 21).

**Conclusion:**

Our observation and the review of the literature reported the possibility of very weak mosaicism and disease‐causing confined tissue‐specific mosaicism in fetus or alive patients with chromosome 21 aneuploidy, mainly Down syndrome. In case of clinical diagnosis suggestive of Down syndrome, attention must be paid to the risk of false‐negative test due to chromosomal mosaicism (very weak percentage, different tissue distribution). To overcome this risk, it is necessary to privilege the diagnostic techniques without culture step and to increase the number of cells and tissues analyzed, if possible. This study highlights the limits of microarray as the unique diagnostic approach in case of weak mosaic and French cytogenetics guidelines recommend to check anomalies seen in microarray by another technique on the same tissue.

## 1 INTRODUCTION

While trisomy 21 or Down syndrome is the most frequent form of chromosomal abnormality in newborns, tetrasomy 21 remains a very rare event. To date, only 12 patients have been reported in the literature and about 42% is represented by a complete tetrasomy. About 2% of clinically diagnosed Down syndrome are mosaic, which represents a real risk of false‐negative prenatal diagnosis especially as the amniotic fluid proportion of trisomic cells can be very low, <10% (Wallerstein et al., 2000).

In this article, we report the first prenatal diagnosis of a mosaic complete tetrasomy 21 with an initially false normal karyotype on amniotic fluid and the second well‐documented clinical description. Positive clinical diagnosis during post‐termination pathologic examination led to molecular cytogenetic analysis permitting secondarily to diagnose mosaic tetrasomy 21.

### 1.1 Clinical Report

It was the second pregnancy from healthy unrelated parents. The first pregnancy had been uneventful. The mother was 39 years old at the time of this second spontaneous conception. The first trimester was unremarkable with an ultrasound examination at 12 weeks of gestation (WG) showing a nuchal translucency at 2 mm for a crown‐rump length at 77 mm. The combined screening test showed a low risk of fetal trisomy 21 (1/2,500).

A second ultrasound at 22 WG showed an isolated complete atrioventricular septal defect (AVSD). Amniotic fluid was sampled for a complete prenatal karyotype. The results were normal with a female karyotype (46,XX) found in 20 metaphases in accordance with the recommendations of the French cytogeneticists society.

Due to the severe heart malformation, termination of pregnancy (TOP) was requested by the parents at 30 WG.

Fetal clinical examination showed broad hands, impression of short arms, and facial features suggestive of trisomy 21: round face, flat profile, hypertelorism, upslanting palpebral fissures, marked suborbital folds, dysplastic ears, and interposed tongue. Cardiac examination confirmed the complete AVSD. X‐ray radiographs of the skeletal system were normal. The histological examination of various tissues did not show any particular element. However, the combination of heart malformation and facial features was strongly suggestive of trisomy 21. Interestingly, it should be noted that both parents have upslanting palpebral fissures.

Because of normal amniotic fluid karyotype, a pangenomic microarray analysis was performed on lung tissue. Additional tissues (lung, liver, and thymus) were examined and genetic markers were studied to complete the cytogenetic studies.

## 2 MATERIALS AND METHODS

Cytogenetic analysis was performed according to standard techniques from cultured amniocytes, using RHG‐banded chromosomes. Fetal karyotype was analyzed using Ikaros software (MetaSystems).

DNA was extracted from fetal tissue (lung, liver, thymus) using QIAamp DNA Mini Kit (Qiagen) according to the manufacturer's protocol.

Chromosomal microarray analysis (CMA) was performed using SurePrint G3 Human CGH Microarray ISCA 60K v2 (Agilent), according to the supplier's instructions. Results were processed and visualized through Cytogenomics 2.0 software (Agilent).

FISH analyses were performed according to the probe provider's protocol on nuclei from amniotic fluid and fetal tissues (lung, liver, thymus) using touch‐preparation slides. The FISH experiments used several sets of probes: a mix of loci‐specific 21q22.13 (D21S270/D21S341) and 13q14.2 (D13S1195/D13S1218) probes (Aquarius, Cytocell), a mix of centromeric probe for chromosome 21 (D21Z1), which cross‐hybridizes with centromere of chromosome 13 (D13Z1), and sub‐telomeric 21qter probe (21q22.3) (Kreatech, Leica Biosystems), and non commercial BAC probe RP11‐31B6 (21q11.2) (Table S1). Hybridizations were analyzed using Isis software (MetaSystems).

We used a multiplex QF‐PCR (Quantitative Fluorescent Polymerase Chain Reaction) method in order to assess copy numbers for chromosomes 21 on fetal tissue DNA using ChromoQuant SuperSTaR Optima kit (Cybergene AB) according to the supplier's recommendations. Fluorescence‐labeled PCR products were electrophoresed in ABI Prism 3130 Genetic Analyzer and analyzed with the GeneMapper software version 5.0 (Thermo Fisher Scientific). The allele ratio for the normal range was from 0.8 to 1.4. If a marker ratio was higher than 1.8 (or lower than 0.65), or more than two peaks were detected for a marker, this marker was presumptive to be at least trisomic. Unfortunately, it was not possible to obtain blood samples from the parents to study parental origin of supernumerary chromosomes 21. Genetic chromosome 21 markers are listed in Table S1.

### 2.1 Ethical Compliance

As required by the French national laws, parents’ consent was obtained for pathological examinations and genetic investigations of the fetus. This study was approved by the hospital ethics committee.

## 3 RESULTS

**Figure 1:**
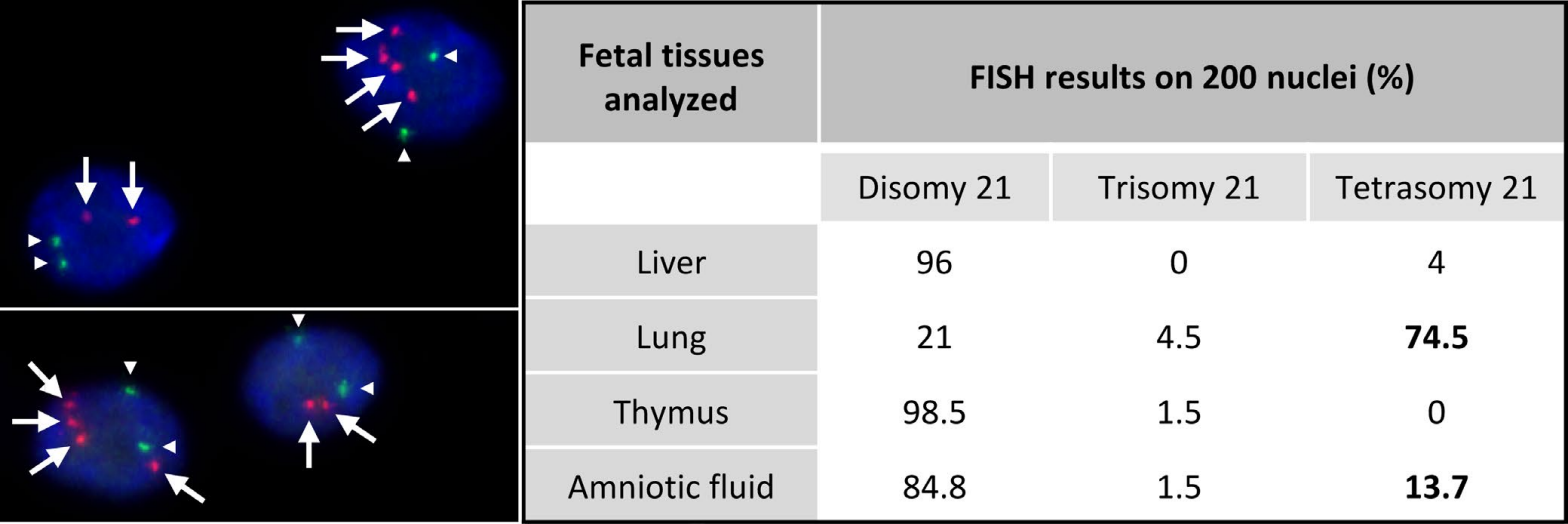
Results of FISH analysis. Left: Two pictures showing specific FISH probes for 13q14.2 locus (signals marked by white triangles) and 21q22.13 locus (signals marked by white arrows) hybridized on lung tissue. DAPI for counterstaining. Mosaicism of tetrasomic cells (four signals for the four copies of chromosome 21 and two signals for two copies of chromosome 13) and disomic cells for chromosome 21 (and chromosome 13). Right: Table showing cell counts for each FISH pattern on fetal tissues and amniotic fluid. Confirmation of mosaic tetrasomy 21 in lung tissue (74.5%) and uncultured amniotic fluid (13.7%) by FISH analysis with a percentage of trisomic 21 cells at the limit of the significance level

**Figure 2:**
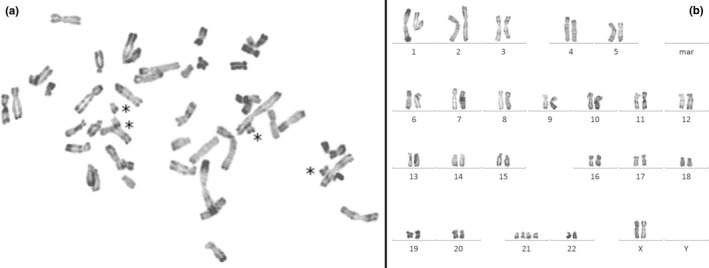
Fetal metaphasis and karyotype on amniotic fluid. Metaphasis (a) and Karyotype (b) with 48 chromosomes (R‐banding) and four free copies of chromosomes 21 (black asterisks), in favor of a free tetrasomy 21

Prenatal standard karyotype on amniotic fluid displayed a normal chromosomal formula 46,XX. CMA on fetal lung DNA showed a significant deviation for all probes of chromosome 21 (Figure S1) with a mean log2 ratio at +0.453 corresponding to mosaic duplication at 74% (Calculation according to [Valli et al., 2011]). For all other chromosomes, ratios were balanced. Based on this result, we suspected a mosaicism of trisomy 21. Surprisingly, FISH analysis displayed a variable number of signals in the tested fetal tissues, ranging from 2 to 4 spots, corresponding to a mosaicism of disomy, trisomy, and tetrasomy of the chromosome 21 (Figure 1 and Figure S2). Lung sample was predominantly tetrasomic with 74.5% of analyzed cells. Other analyzed tissues (liver, thymus) were mainly disomic for chromosome 21. We failed to analyze by FISH the fixed heart tissue. Despite a normal prenatal karyotype, complementary FISH analysis on uncultured amniotic fluid cells of initial prenatal sampling showed 13.7% of tetrasomic cells. All the FISH results on the different tested tissues are summarized in Figure 1. After CMA results, we reanalyzed the amniotic fluid of prenatal diagnosis by studying 50 additional metaphases and this analysis revealed the presence of two metaphases with four free chromosomes 21 (Figure 2). Due to the limitations of FISH techniques (superposition of signals and/or split signal), cells with a three‐signal pattern representing <5% of observed cells were not interpreted as trisomic cells (Pujol et al., 2004). With this notion of tetrasomy, the log2 ratio value by the CMA gives a mosaic at 37% (Calculation according to [Valli et al., 2011]). To genetically characterize the chromosomes 21, we studied by QF‐PCR with eight genetic markers all along the chromosome 21 on lung, thymus, and liver DNA (Table S1). On the lung DNA, two markers showed the presence of three alleles with roughly equivalent area under the curve, and six markers only two alleles, one with a larger area under the curve, in comparison with the two alleles seen on the DNA of other tissues (Figure S3). With the notion of tetrasomy, the area under the curve allowed to calculate a mosaic rate around 41%. In conclusion, the fetus had a mosaic of disomic and tetrasomic cells for chromosome 21.

## 4 DISCUSSION

In this report, isolated complete AVSD was the only feature in favor of a trisomy 21 during the prenatal period. AVSDs are the diseases most often associated with a chromosomal abnormality. About 50% of patients with AVSD have trisomy 21 and AVSDs account for about 50% of congenital heart diseases in Down syndrome patients (Pfitzer et al., 2018). Prenatal investigations such as maternal serum screening and amniotic standard karyotype failed to reveal any aneuploidy or other chromosome abnormality. After TOP, although first trimester prenatal screening and initial fetal karyotype were normal, the cardiac abnormality and the characteristic face of the fetus questioned us again on the diagnosis of a trisomy 21. The CMA arbitrarily performed on lung tissue DNA was in favor of a mosaicism of complete trisomy 21 at 74% (Mean log2 ratio = +0.453 for chromosome 21). Surprisingly, FISH realized in lung tissue to confirm a trisomy 21 revealed a tetrasomy 21 with a high proportion of cells with four copies of chromosomes 21. The same FISH analysis performed on other fetal tissues showed mainly disomic cells for chromosome 21 (Figure 1). FISH on amniotic fluid displayed a significant rate (13.7%) of nuclei with four chromosomes 21 although the advanced karyotype revealed only two tetrasomic 21 cells on 50 analyzed cells (4%) (Figure 2). QF‐PCR showed three alleles for two genetic markers and two alleles with an uneven area under the curve for six markers only on lung tissue DNA, signaling the presence of at least three chromosomes 21. In summary, molecular analyses could evoke a trisomy 21 but cytogenetic analyses diagnosed a tetrasomy 21. This discrepancy in the results could be explained by difficulties for chromosomally abnormal cells entering into cell division, especially if a long‐term culture could have selected normal clones (Persutte & Lenke, 1995; Reeser & Wenger, 1992). These observations suggest that mosaicism abnormalities would be better diagnosed using techniques without culture. In the same way, there was a difference between the evaluation of the mosaic rate by FISH (74.5%), CMA (37%) and genetic markers (41%). This difference could be explained because the FISH technique using touch‐preparation slides explores a localized tissue region that may contain a predominantly tetrasomic cell line whereas the DNA used in the other techniques results from a mixture of several not all‐tetrasomic cell lines from a larger tissue volume. Assuming this, the tetrasomy 21 was initially homogeneous occurring in a meiotic event and the observed mosaicism might be due to a natural selection of normal cells, as has been demonstrated for Pallister‐Killian mosaicism (Schubert, Viersbach, Eggermann, Hansmann, & Schwanitz, 1997) or a “tetrasomy” rescue in preimplantation embryo culture (Munné et al., 2005).

Tetrasomies 21 are very rare events. To date, only 12 patients with tetrasomy 21 have been reported in the literature including seven cases of partial tetrasomy and five cases of complete tetrasomy (Table S2). For the seven cases of partial tetrasomy 21, the most frequent features are neonatal hypotonia, intellectual disability, and development/speech delays. Most of them present morphological changes of the skull like microcephaly or brachycephaly. Concerning the face, tongue is mostly large and/or protruding in connection with hypotonia. At last, the majority of patients showed brachydactyly. There are no cardiac or digestive malformations. These findings are usually encountered in patients with trisomy 21 (Weijerman & de Winter, 2010). Most authors consider that the phenotype is that of atypical trisomy 21 associating one or more signs described in trisomy 21 with nonspecific abnormalities (Capkova, Misovicova, & Vrbicka, 2014; Cerretini et al., 1999; Daumer‐Haas et al., 1994; Gutiérrez‐Angulo, Ramos, Dávalos, Sánchez‐Corona, & Rivera, 1999; Nagarsheth & Mootabar, 1997; Rost et al., 2004; Slavotinek et al., 2000). These cases show an additional isodicentric chromosome 21 not including the Down Syndrome critical region (21q22.2q22.3).

For the five cases of complete tetrasomy 21, four have been diagnosed prenatally with no more clinical information and unprecised outcome for three of them (Hahnemann & Vejerslev, 1997; Liehr et al., 2001; Soler et al., 1999), of which one in mosaic with trisomy 21 (Hahnemann & Vejerslev, 1997). Best, Brooks, & Clarkson, (1996) described a mosaic confined to the placenta with the birth of a healthy child without development abnormality afterward. To date, only one case of homogeneous complete tetrasomy 21 on blood cells was diagnosed postnatally (Jabs, Stamberg, & Leonard, 1982). The authors reported on a newborn boy with Down syndrome phenotype and congenital monocytic leukemia but this condition was challenged because the presence of two supernumerary chromosomes 21 is a relatively frequent event in leukemic cells (Heerema et al., 2007; Liehr et al., 2001). It was also mentioned a patient with Down syndrome and acute megakaryoblastic leukemia associated with a pentasomy 21q with two isochromosomes (Park et al., 2015). In our study, we consider that tetrasomy 21 is not related to underlying leukemia. We do not have a mosaic trisomic cell population in the analyzed tissues. The ultrasound and pathological examinations with the used techniques do not show any element in favor of possible prenatal leukemia, such as hepatosplenomegaly, various effusions, and fetal hydrops in the third trimester (Fouché et al., 2010). Finally, the clinical expression of tetrasomy 21 could be that of trisomy 21, but more severe. This corresponds to the commonly accepted fact that four copies of a chromosome lead to a similar or a more severe phenotype than a trisomy (Schinzel, 1993).

Three cases were reported during prenatal diagnosis for advanced maternal age whereas ultrasound examinations were normal. For two fetuses, a tissue‐specific mosaicism of the chromosome abnormality was observed highlighting cytogenetic instability for tetrasomy 21 (Nagarsheth & Mootabar, 1997; Soler et al., 1999). Therefore the low proportion of abnormal cells represents a risk of misdiagnosis if the number of analyzed cells is not sufficient.

Concerning the chromosome mechanisms at the origin of tetrasomy 21, we consider only patients with molecular confirmations (FISH and/or CMA) of the chromosome abnormality. All the cases that could be studied are de novo.

For the cases of partial tetrasomy, an isodicentric chromosome was found: a supernumerary marker chromosome composed of the duplication of the proximal part of the long arm of a chromosome 21. These markers may be the consequence of a U loop mechanism (Slavotinek et al., 2000) and led to a partial tetrasomy of the involved chromosome. As described in 12p tetrasomy, isochromosomes are unstable and may be lost during cell division explaining the mosaicism (Reeser & Wenger, 1992). Moreover, four other patients have been reported but were confused with mosaic tetrasomy 12p due to use of banding techniques alone (Gilgenkrantz et al., 1987; Hall, 1985; Nagarsheth & Mootabar, 1997). This may also be the case of a little girl who died shortly after birth in a context of severe malformations without Down syndrome phenotype and in whom a weak mosaic of a hexasomy 21 (with two supernumerary marker chromosomes supposed to be a double 21;21 translocation) on amniotic fluid and skin but not on blood was demonstrated (Ketupånyå, Crandåll, Blanchard, & Rogers, 1984).

In the case of complete tetrasomy, the causal mechanism could be a maternal double nondisjunction in meiosis I and II (Liehr et al., 2001) or the combination of both maternal and paternal meiotic nondisjunctions of chromosomes 21 (Figure S4).

Chromosomal studies on human gametes and preimplantation embryos have provided valuable data on the process of chromosomal segregation and mosaicism occurrence. Mosaicism may originate from a variety of mechanisms including chromosome missegregation, anaphase chromosome lagging, endoreplication, tripolar mitosis, micronuclei formation, or chromatid predivision (Ottolini et al., 2017; Taylor et al., 2014). Furthermore, mosaicism can be trigged by any one of numerous factors such as defective sister chromatid cohesion, spindle instability, centrosome dysfunction, weakness of mitotic checkpoints, or aging (Capalbo, Hoffmann, Cimadomo, Ubaldi, & Rienzi, 2017; Greaney, Wei, & Homer, 2017; Ly, Lockhart, Lerner, & Schultz, 2000; Pihan, 2013; Vázquez‐Diez & FitzHarris, 2018). Chromosomal mosaicism is prevalent throughout human preimplantation development (Lebedev, 2011). It seems that aneuploidies and mosaicisms are frequent during the first cleavage divisions (up to 80% of cells affected) and then decrease significantly (30–40%) during blastocyst maturation (Fragouli et al., 2013; Johnson et al., 2010; Santos et al., 2010; Vera‐Rodriguez & Rubio, 2017). These data are consistent with the assumption of self‐correction of chromosomally abnormal embryos, based on the trisomy rescue process (Munné et al., 2005). Moreover, the reduced proliferation of aneuploid cells into an embryo could also explain the vanishing of mosaicism. Mouse model of preimplantation chromosome mosaicism revealed the gradual elimination of abnormal cell by apoptosis, then allowing the mosaic embryos to have a comparable development potential than normal embryos (Bolton et al., 2016). However, the fate of aneuploid cells seems to depend on the lineage, and on when de novo mitotic chromosomal missegregation or trisomy rescue occur during the embryonic development (Mantzouratou & Delhanty, 2011).

Advances in single‐cell genome sequencing technologies should provide further insight into the mechanism and impact of mosaicism. Thus, the study of crossovers and chromosome segregation patterns in human oocytes and corresponding polar bodies, named MeioMapping, has led to the discovery of new type of chromosome segregation error, the reverse segregation, susceptible to induce preimplantation chromosome mosaicism (Ottolini et al., 2015). Also, the single cell analysis of copy number variations in different human tissues supported the concept that early embryonic chromosome instability might result in stable mosaic pattern in human tissues (Mkrtchyan et al., 2010). In the present case, the comprehensive analysis of genetic markers such as microsatellites could have made it possible to predict the meiotic or mitotic nature of the chromosomal mosaicism, as well as the parental origin of the supernumerary chromosomes 21. Unfortunately, it was not possible to obtain parental blood samples to perform this type of analysis.

The present case and the review of literature raise the issue of the possible failure to detect weak but true pathological mosaicism in prenatal diagnosis. In front of atypical or mild clinical findings of Down syndrome, we suggest to perform karyotype analysis on more cells than recommended or usually practiced. FISH analysis with chromosome 21‐specific probes on uncultured amniotic fluid or other available tissues could be very useful for a complementary study. We stress that CMA performed directly on DNA from uncultured amniotic fluid could be interpreted cautiously considering the risk of mosaicism.

## CONFLICT OF INTEREST

The authors declare no conflict of interest.
