# Supplementary material for: Mosaic complete tetrasomy 21 in a fetus with complete atrioventricular septal defect and minor morphological variations
Source: Mol Genet Genomic Med. 2019 Sep 7;7(11):e00895. doi: 10.1002/mgg3.895 (PMC6825868; doi:10.1002/mgg3.895)
Supplement: Supplementary file 1 [file MGG3-7-e00895-s001.pdf]

| | Names | Cytogenetic Location | Markers | Genes or sequence positions (3) |
|---|---|---|---|---|
| FISH probes | CEP13/21 (1) | 21p11.1-q11.1 | D21Z1 | Centromere |
| | RP11-31B6 | 21q11.2 | SHGC-83252/SHGC-34996 | 16076691-16249383 |
| | 21 unique sequence (2) | 21q22.13 | D21S270/D21S341 | DIRK1A, KCNJ6, DSCR4, DRCS8 |
| | Sub-telomere 21qter | 21q22.3 | D21S1446 | DIP2A, S100B |

| | | | | |
|---|---|---|---|---|
| Genetic markers (4) | D21S11 | 21q21.1 | D21S11 | 20554259-20554481 |
| | D21S1437 | 21q21.1 | D21S1437 | 21646683-21647003 |
| | D21S1409 | 21q21.2 | D21S1409 | 24348717-24349076 |
| | D21S1435 | 21q21.3 | D21S1435 | 27848841-27849143 |
| | D21S1442 | 21q21.3 | D21S1442 | 28818455-28818753 |
| | D21S2052 | 21q21.3 | D21S2052 | 28818565-28818853 |
| | D21S1444 | 21q22.13 | D21S1444 | 39359577-39359893 |
| | D21S1246 | 21q22.2 | D21S1246 | 40870994-40871546 |

(1) Cross-hybridization with alpha-satellite centromeric region 13p11.1-q11.1 (D13Z1)
(2) Co-hybridization with probe 13 unique sequence (13q14.2 - D13S1195/D13S1218)
(3) All nucleotides positions are according with the GRCh37/hg19 human reference sequence
(4) Genetic markers of chromosome 21 from ChromoQuant SuperSTaR Optima kit (CyberGene AB, Solna, Sweden)

Data from commercial suppliers and public databases (Ensembl, UCSC)

**<u>Table S1:</u>** List of FISH probes and genetic markers of chromosome 21 covering all chromosome 21 regions.

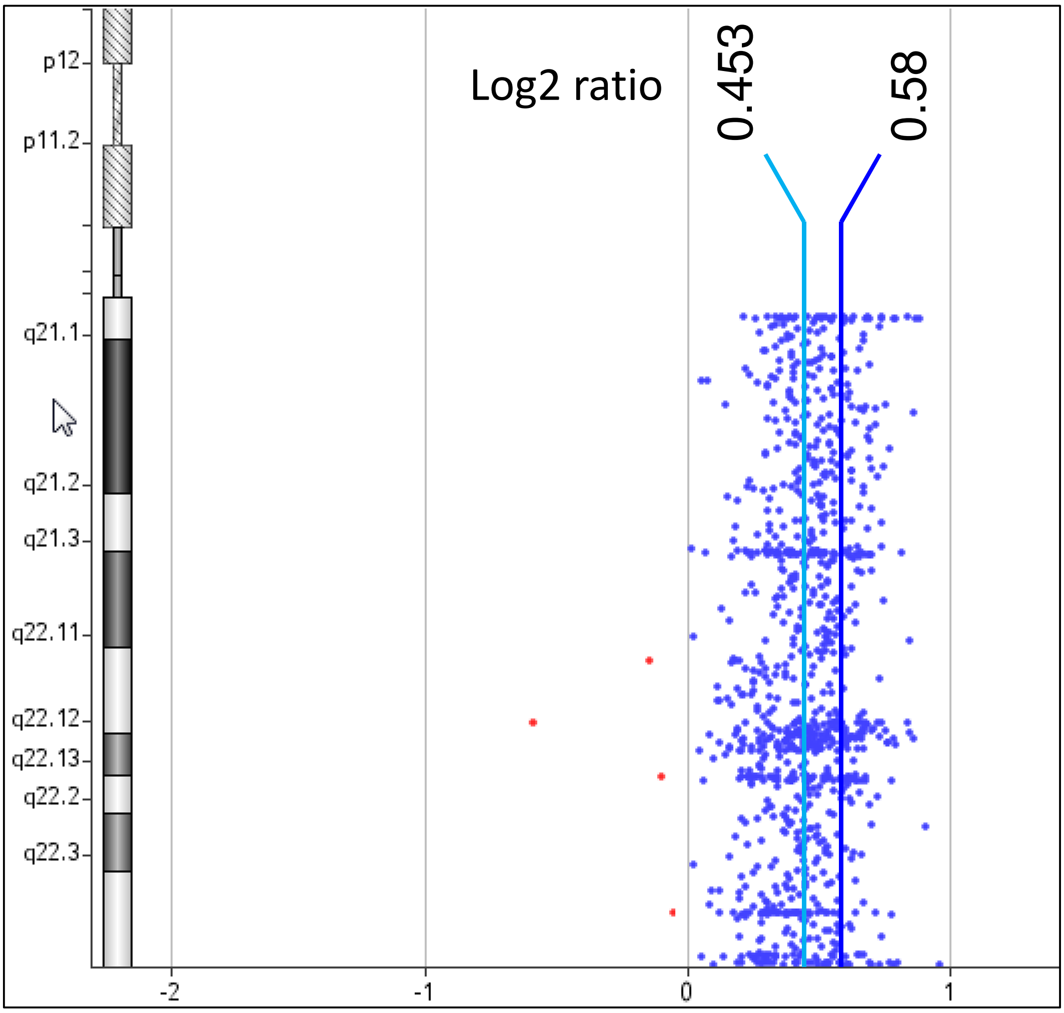


**Figure S1:** CMA hybridization profile of fetal DNA extracted from lung tissue for the chromosome 21.

Nearly all the chromosome 21 probes (dark blue) are deviated in the direction of complete duplication with a mean log2-ratio value of 0.453 (light blue line) reflecting a chromosomal mosaic. The log2-ratio value of 0.58 corresponds to homogeneous complete duplication (dark blue line). The x-axis represents the log2 ratio (Number 0 represents 2 copies or disomy, number 1 represents 4 copies or complete tetrasomy), the y-axis the different regions of chromosome 21 from telomere short arm (top) to telomere long arm (bottom).

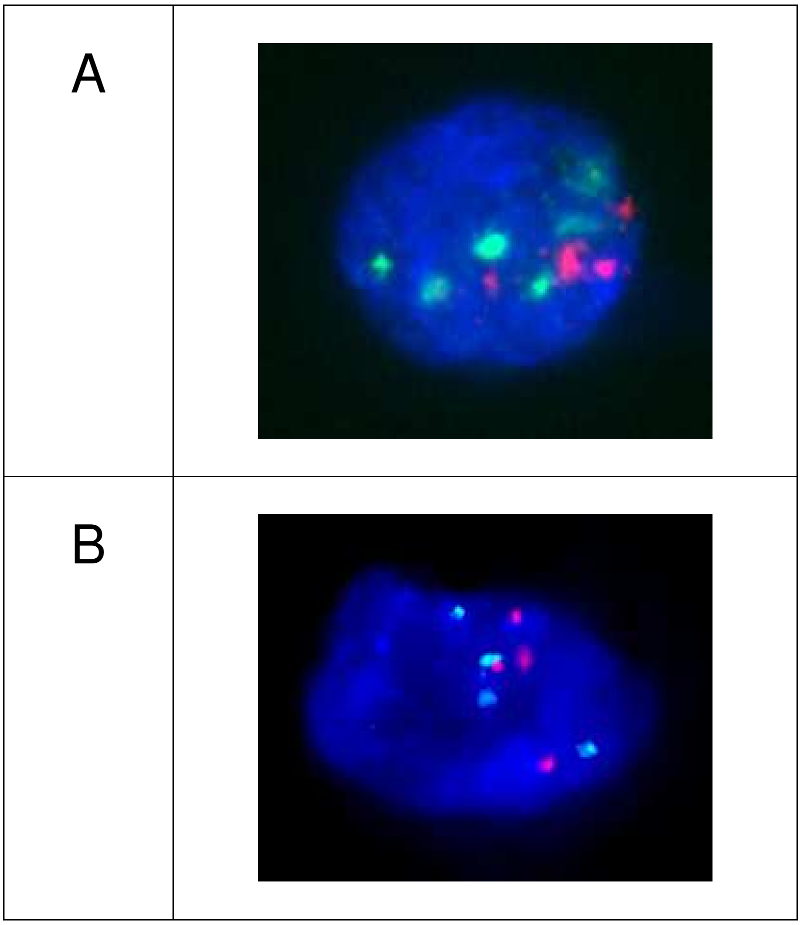


**Figure S2:** FISH on touch-preparation slide of lung tissue

A: Hybridization showing 6 green signals for CEP13/21 probe (4 for chromosome 21 and 2 for chromosome 13) and 4 red signals for sub-telomere 21qter, in favor of a tetrasomy 21.

B: Hybridization showing 4 red signals for RP11-31B06 probe (21q11.2) and 4 blue signals for sub-telomere 21qter.

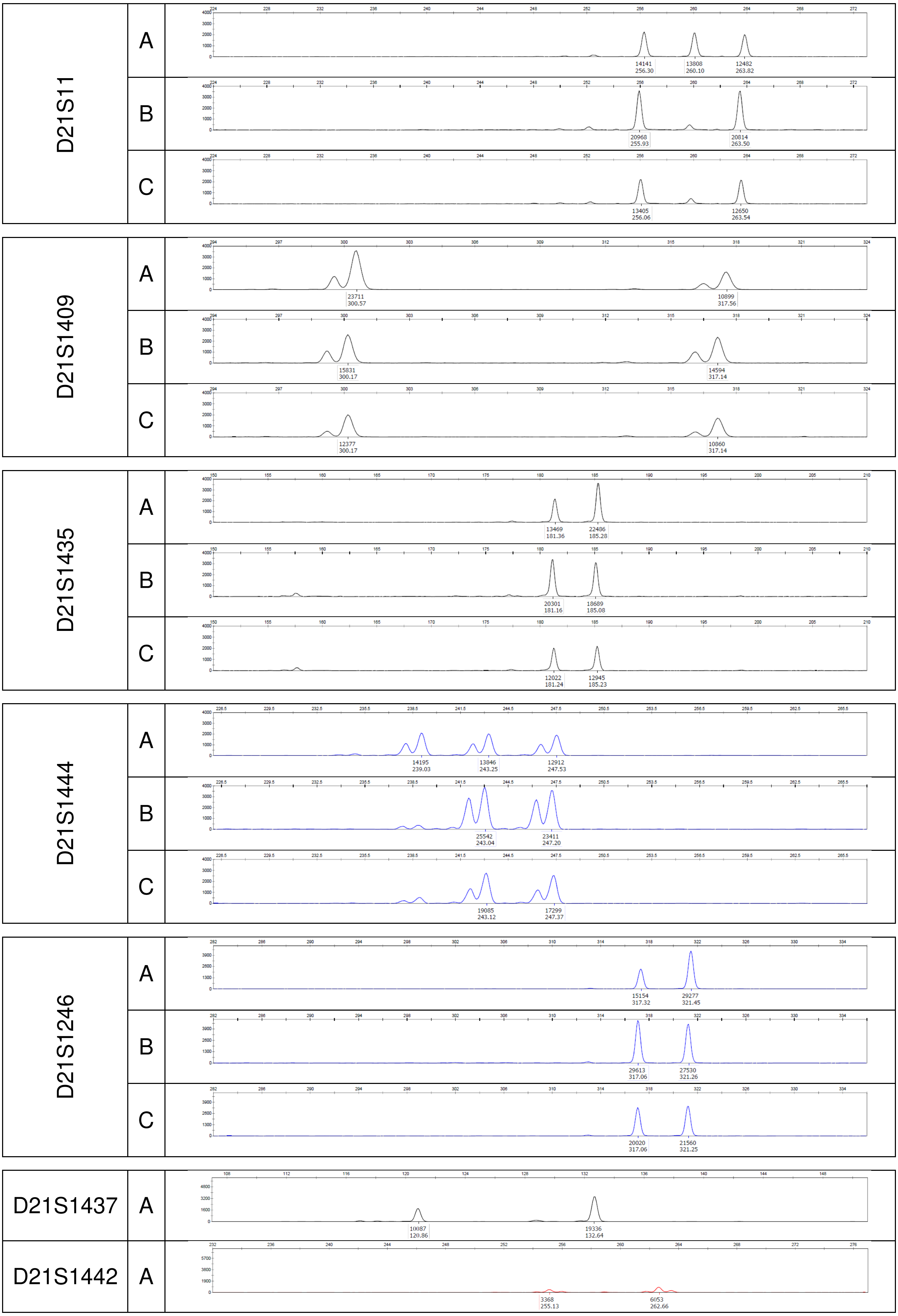
D21S11
A
14141 256.30
13808 260.10
12482 263.82
B
20968 255.93
20814 263.50
C
13405 256.06
12650 263.54
D21S1409
A
23711 300.57
10899 317.56
B
15831 300.17
14594 317.14
C
12377 300.17
10860 317.14
D21S1435
A
13469 181.36
22486 185.28
B
20301 181.16
18689 185.08
C
12022 181.24
12945 185.23
D21S1444
A
14195 239.03
13846 243.25
12912 247.53
B
25542 243.04
23411 247.20
C
19085 243.12
17299 247.37
D21S1246
A
15154 317.32
29277 321.45
B
29613 317.06
27530 321.26
C
20020 317.06
21560 321.25
D21S1437
A
10087 120.86
19336 132.64
D21S1442
A
3368 255.13
6053 262.66

| | | |
|---|---|---|
| D21S2052 | A | 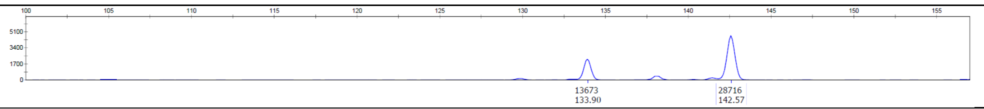  |

**Figure S3:** Migration profile of PCR products corresponding to amplified genetic markers from DNA of different fetal tissues (A: lung, B: thymus, C: liver).

For the lung, the genetic markers present 3 alleles for markers D21S11, and D21S1444 with areas under the curve roughly equal and 2 alleles for markers D21S1409, D21S1435, and D21S1246 with each time one of the two alleles with an area under the curve greater than that of the second allele. For the thymus and liver, the genetic markers present 2 alleles for all markers with an area under the curve roughly equal for both. Genetic markers (D21S1437, D21S1442, D21S2052) only tested on the lung also show a higher area under the curve for one of the 2 alleles (At the bottom).

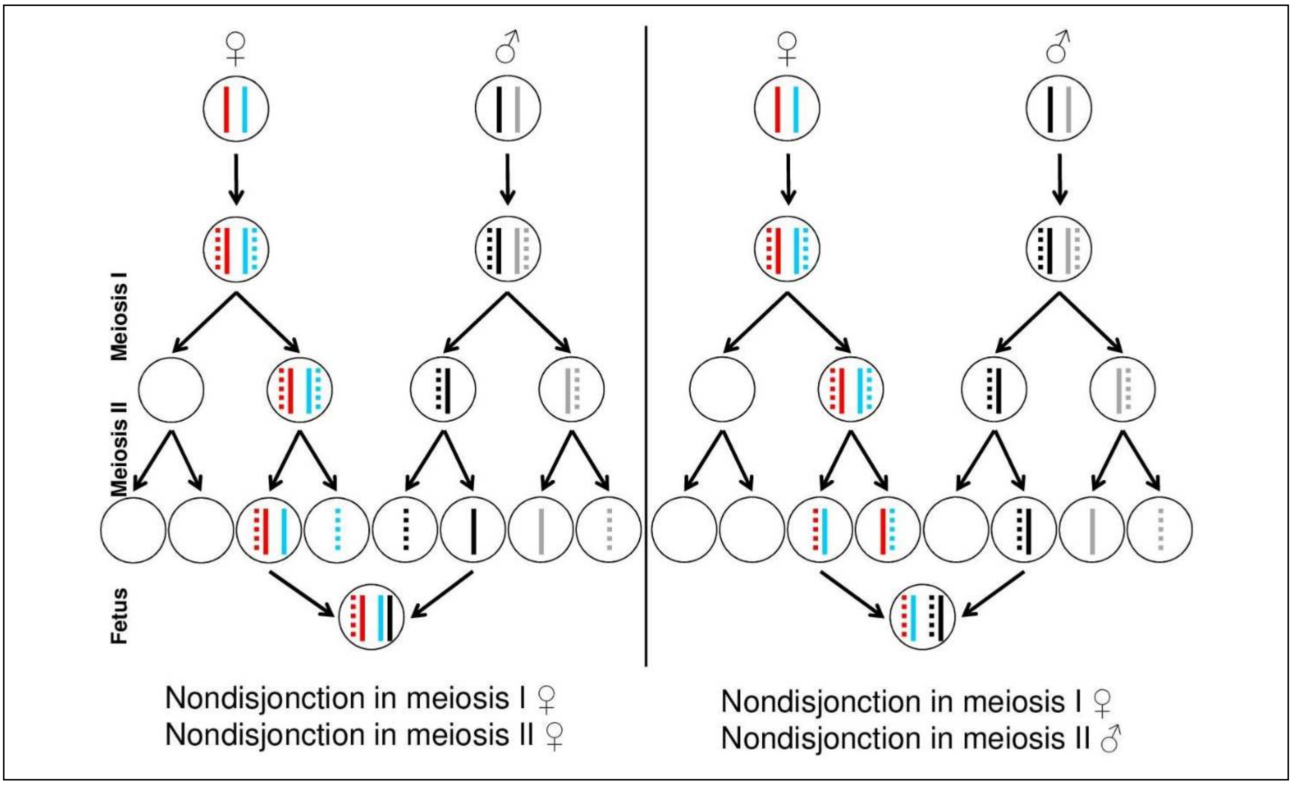


**Figure S4:** Diagram illustrating the possible mechanism of nondisjunction in chromosome 21

The nondisjunction occurs during the gametogenesis and can lead to a complete tetrasomy 21 after failure to separate homologous chromosomes (Meiosis I) or sister chromatids (Meiosis II) (either two successive nondisjunctions during oogenesis (left), or a nondisjunction in meiosis I during oogenesis associated with a nondisjunction in meiosis II during spermatogenesis (right)).
