# Supplementary material for: Mosaic complete tetrasomy 21 in a fetus with complete atrioventricular septal defect and minor morphological variations
Source: Mol Genet Genomic Med. 2019 Sep 7;7(11):e00895. doi: 10.1002/mgg3.895 (PMC6825868; doi:10.1002/mgg3.895)
Supplement: Supplementary file 2 [file MGG3-7-e00895-s002.pdf]

PARTIAL TETRASOMY

Postnatal diagnosis

	Case Report	Capkova P et al. 2014	Rost I et al. 2004	Slavotinek AM et al. 2000	Gutiérrez-Angulo M et al. 1999	Cerretini R et al. 1999	Daumer-Haas C et al. 1994
	Antenatal history	uneventful	uneventful	IUGR (30WG) Birth at 33 WG	uneventful	uneventful	uneventful
	Maternal / Paternal Age	31 / ?	33 / 40	34 / 33	33 / 32	46 / 52	32 / ?
Cytogenetics investigations	Chromosome abnormality [Cell number]	* 47,XY,+psu idic(21)(q21) (case 2)	47,XY,+psu idic(21)(q21.2)	47,XY,+psu idic(21)(q21.1) [30] (blood and skin)	47,XY,+psu idic(21)(q22.1) [50]	47,XY,+psu dic(21)(q22.11) [100]	47,XX, +idic(21)(q22.1) [100]
	DSCR 21q22.2q22.3 (FISH)	- (CMA)	- (CMA)	-	-	-	-
	Molecular confirmation	FISH / CMA	FISH / CMA	FISH	FISH	FISH	FISH
	Mosaïcism	no	no	no	no	no	no
Neurology	Neonatal hypotonia	+	+	-	+		+
	Truncal hypotonia			mild			
	Intellectual disability	-	+		+	+	+
	Speech delay	+	+		+	+	+
	Developmental delay	+	+	+	+	+	+
	Hyporeactivity				+		
	Joint hyperlaxity	+			+	-	+
	Skin hyperelasticity				+		
	Seizures			absences			
	Brain CT-scan			cerebral hypodevelopment	N	N	
Craniofacial features	Microcephaly	-	+	+	+	-	+
	Skull Anormal Shape	-	-	+	+	+	+
	Prominent Forehead				+		
	Round face	-	+			+	+
	Bilateral Eye Ptosis			+			
	Hypertelorism			+			
	Canti abnormalities			+	+	-	-
	Strabism			+			
	Palpebral fissures	N	upslanting	non-slanting	downslanting	horizontal	short / upslanting
	Brushfield spots	-	+		+	-	-
	Midface hypoplasia			+	+		
	Nasal bridge	N	flat	wide		high	
	Mouth		open	small, triangular	open	prominent lower lip	
	Tongue	large		large, protruding	protruding	N	large, protruding
	Philtrum			short			
	Chin			small			
	Short neck / Excess skin			+ / +	- / -	- / -	+ / -
	Ears	N	N	pointed helices	large	N	N
	Third fontanel					-	+
Extremities	Brachydactyly / Clinodactyly	- / -	5th finger / -	+ / 5th finger	2nd-5th toes / -	- / -	+ / -
	Hands / Thumbs	N		short / short		N	short, broad
	Toenails			hypoplastic			
	Dermatoglyphic features	N	ulnar loops		ulnar loops distal axial triradius	N	ulnar loops distal axial triradius
	Single transverse palmar crease	-	-	-	-	-	+
	Feet			small			
	Sandal Gap	+	+		-	-	-
Abdominal features	Umbilical hernia			+		-	+
	Genitalia			hypospadias micropenis shawl scrotum		macrogenitalism	
	Gastrointestinal tract	N			N	N	N
	Diastasis recti					-	+
Others	Short stature	-	+	+	-	-	-
	Pectus excavatum				+		
	Cardiac examination	N	N	N	N	N	N
	Others			strawberry naevi high anterior hairline widow's peak bilateral ptosis hypertelorism	no skeletal X-ray abnormalities	precocius puberty	

Prenatal diagnosis

		Case Report	Nagarsheth NP et al. 1997
		Medical indication	advanced maternal age
		Outcome	delivery at 38 WG
		Maternal / Paternal Age	38 / ?
Prenatal samples		Amniotic fluid	mos47,XY,+i(21q)[6]/ 46,XY[9]
		Direct CVS	
		Cultured CVS	
		Other	cord blood: 46,XY[43]
Postnatal samples		Blood	+i(21q)[1/120]
		Fibroblast	
		Molecular confirmation	FISH
		Mosaïcism	yes
		Neurology	no hypotonia
		Craniofacial features	frontal bossing mild brachycephaly micrognathia open sutures ocular Brushfield spots right epicanthal fold depressed nasal bridge increased postnuchal skin
		Extremities	bilateral 5th clinodactyly sandal gap
		Abdominal features	
		Other	skin peeling and split normal cardiac anatomy

COMPLETE TETRASOMY

Postnatal diagnosis

	Case Report	Jabs EW et al. 1982
	Antenatal history	delivery at 32 WG
	Maternal/Paternal Age	24 / 36
Cytogenetics investigations	Chromosome abnormality [Cell number]	48,XY,+21,+21[50]
	Molecular confirmation	no
	Mosaïcism	no
Neurology	Neonatal hypotonia	+
	Truncal hypotonia	
	Intellectual disability	
	Speech delay	
	Developmental delay	
	Hyporeactivity	
	Joint hyperlaxity	+
	Skin hyperelasticity	
	Seizures	
	Brain CT-scan	
Craniofacial features	Microcephaly	
	Skull Anormal Shape	+
	Prominent Forehead	
	Round face	
	Bilateral Eye Ptosis	
	Hypertelorism	
	Canti abnormalities	+
	Strabism	
	Palpebral fissures	small / upslanting
	Brushfield spots	
	Midface hypoplasia	
	Nasal bridge	flat
	Mouth	
	Tongue	
	Philtrum	
	Chin	
	Short neck / Excess skin	+ / +
	Ears	small, unfolded helix
	Third fontanel	
Extremities	Brachydactyly / Clinodactyly	? / -
	Hands / Thumbs	
	Toenails	
	Dermatoglyphic features	
	Single transverse palmar crease	-
	Feet	
	Sandal Gap	+
Abdominal features	Umbilical hernia	
	Genitalia	
	Diastasis recti	
Others	Short stature	
	Pectus excavatum	
	Cardiac examination	PDA
	Others	bilobed right lung hypoplastic pelvis hepatosplenomegaly congenital monocytic leukemia kernicterus died at 4 days of life

Prenatal diagnosis

		Case Report	This study	Liehr T et al. 2001	Soler A et al. 1999	Hahnemann JM and Vejerslev LO, 1997	Best RG et al. 1996
		Medical indication	cardiac abnormality on ultrasound	spontaneaous abortion at 13 WG	advanced maternal age		advanced maternal age
		Outcome	TOP at 30 WG		TOP at 13 WG		late preeclampsia delivery at 39 WG
		Maternal/Paternal Age	39 / 36.5	34 / ?	40 / ?	? / ?	38 / ?
Prenatal samples		Amniotic fluid	48,XX,+21,+21[2]/46,XX[48] FISH: tetra 21(13.7%)				46,XY[55]
		Direct CVS			48,XY,+21,+21[10]/ 47,XY,+21[10]	+21/+21,+21	46,XY
		Cultured CVS		47,XY,t(14;21),+21[15]	47,XY,+21		48,XY,+21,+21[8]/46,XY[7]
		Other	lung: tetra 21(74.5%)		cord blood: 46,XY		
Postnatal samples		Blood					46,XY[50]
		Fibroblast		47,XY,t(14;21),+21[15]	46,XY (kidney: tri 21(19%))		46,XY[55]
		Molecular confirmation	FISH / CMA	FISH	FISH		FISH
		Mosaïcism	yes	no	yes	yes	confined to the placenta
		Neurology		No clinical informations	No clinical informations	No clinical informations	Healthy child, no phenotypical and developmental anomaly at 11 months of life
		Craniofacial features	round face flat profile dysmorphic ears hypertelorism upslanting palpebral fissures marked suborbital folds tongue interposition				
		Extremities	broad hands				
		Abdominal features					
		Other	complete AVSD				

* triplication 21q11.2 (~4 Mb) + duplication 21q11.2q21.3 (~15 Mb) on aCGH (Capkova et al., 2014)

Table S2: Clinical and cytogenetic features of published report cases with tetrasomy 21.

"Partial tetrasomy" indicates that only a part of chromosome 21 is triplicated. The mechanism is therefore a structural abnormality of the involved chromosomes, such as a supernumerary isochromosome. "Complete tetrasomy" indicates that all fragments of a chromosome are triplicated. Different kinds of abnormalities can explain it, such as standard tetrasomy (4 free chromosomes) or a supernumerary Robertsonian chromosome.

+ = present (feature)
- = absent (feature)
? = no information
N = normal examination
+21 = free supernumerary chromosome 21
i(21q) = isochromosome derived from chromosome 21
idic = dicentric isochromosome derived from chromosome 21
rob(14;21) = robertsonian translocation between chromosomes 14 and 21
tetra = tetrasomy
tri = trisomy

CMA = Chromosomal Microarray Analysis
AVSD = AtrioVenticular Septal Defect
CT-scan = Computed Tomography-scan
CVS = Chorius Villi Sampling
DSCR = Down Syndrome Critical Region
FISH = Fluorescence In Situ Hybridization
IUGR = Intra Uterine Growth Retardation
PDA = Patent Ductus Arteriosus
TOP = Termination Of Pregnancy
WG = Weeks of Gestation

psu = pseudo
